# Optimal Representation of Anuran Call Spectrum in Environmental Monitoring Systems Using Wireless Sensor Networks

**DOI:** 10.3390/s18061803

**Published:** 2018-06-03

**Authors:** Amalia Luque, Jesús Gómez-Bellido, Alejandro Carrasco, Julio Barbancho

**Affiliations:** 1 Ingeniería del Diseño, Escuela Politécnica Superior, Universidad de Sevilla, 41004 Sevilla, Spain; jesgombel@outlook.es; 2 Tecnología Electrónica, Escuela Ingeniería Informática, Universidad de Sevilla, 41004 Sevilla, Spain; acarrasco@us.es; 3 Tecnología Electrónica, Escuela Politécnica Superior, Universidad de Sevilla, 41004 Sevilla, Spain; jbarbancho@us.es

**Keywords:** environmental monitoring, audio monitoring, sensor network, sound classification

## Abstract

The analysis and classification of the sounds produced by certain animal species, notably anurans, have revealed these amphibians to be a potentially strong indicator of temperature fluctuations and therefore of the existence of climate change. Environmental monitoring systems using Wireless Sensor Networks are therefore of interest to obtain indicators of global warming. For the automatic classification of the sounds recorded on such systems, the proper representation of the sound spectrum is essential since it contains the information required for cataloguing anuran calls. The present paper focuses on this process of feature extraction by exploring three alternatives: the standardized MPEG-7, the Filter Bank Energy (FBE), and the Mel Frequency Cepstral Coefficients (MFCC). Moreover, various values for every option in the extraction of spectrum features have been considered. Throughout the paper, it is shown that representing the frame spectrum with pure FBE offers slightly worse results than using the MPEG-7 features. This performance can easily be increased, however, by rescaling the FBE in a double dimension: vertically, by taking the logarithm of the energies; and, horizontally, by applying mel scaling in the filter banks. On the other hand, representing the spectrum in the cepstral domain, as in MFCC, has shown additional marginal improvements in classification performance.

## 1. Introduction

### 1.1. Environmental Monitoring of Anuran Calls as Indicators of Climate Change

In recent years, the number of devices focused on the monitoring and analysis of environmental parameters has grown strongly. However, the intended purpose is seldom related to the direct measurement of a parameter, and requires the analysis of complex phenomena. An example of this approach is phenology, which deals with the study of periodic plant and animal life cycles, and how some events are related to seasonal and climate variations [1] and, therefore, to global warming. A further example is provided by environmental monitoring operations, such as the use of the wildfire acoustic emission spectrum as the indicator of the type of forest fire [2].

One of the well-known consequences of climate change is its impact on the development of basic physiological functions of various species [3,4,5,6,7], such as the sound produced in the mating call, which plays a central role in sexual selection and reproduction of numerous ectothermic species (those that regulate their temperature from ambient temperature), including Anura (frogs and toads), fish, and insects [8,9,10]. Various acoustic patterns are employed to attract potential mates, to ward off opponents, and to respond to the risks of predation. These sounds are therefore critical to the adaptation of individuals to the environment.

However, sound production in ectotherms is strongly influenced by the ambient temperature [11,12,13,14,15,16,17,18], which can also affect various features of the acoustic communication system. In fact, once the ambient temperature exceeds a certain threshold, then this threshold can restrict the physiological processes associated with the production of the sound, and may even inhibit behaviour calls. As a result, the temperature may significantly affect the patterns of calling songs by modifying the beginning, duration, and intensity of calling episodes and, consequently, influence anuran reproductive activity.

The analysis and classification of the sounds produced by certain animal species have revealed them to be a potentially strong indicator of temperature fluctuations and therefore of the existence of climate change. The results provided by anuran sound analysis [19] are especially interesting.

However, these studies have to be supported by a large number of audio recordings, which are usually collected in the field, and individually analysed at a later time. Fortunately, the emergence of the Wireless Acoustic Sensor Networks (WASN) [20] has changed this approach, although the classification of bio-acoustic sounds remains a very burdensome task. It is estimated that, on average, an expert requires 2 min of listening to identify a species in 1 min of audio [21], thereby rendering it impractical to manually analyse the large volumes of acoustic data provided by modern sensor networks. For this reason, it is imperative to develop intelligent systems that simplify, automate and speed up the task of analysing and labelling sound recordings. An up-to-date review of such systems can be found in [22].

### 1.2. Previous Work

Our research group has been working for several years on the problem of classifying anuran sounds as indicators of global warming, and have enjoyed a long experience of collaboration in the Spanish Doñana National Park where a Sensor Network has been deployed for various purposes.

In a first contribution [23], it was demonstrated that it is possible to automatically classify open-air recorded anuran sounds. In that work, 64 sound records of three different classes were featured using 18 MPEG-7 parameters, whereby two simple classifiers (minimum distance and maximum likelihood) were employed that obtained results of good accuracy. However, in order to attain those good outcomes, ad hoc tuning had to be performed on the proposed standard classifiers, which caused two main drawbacks: the analysis procedure had to be adapted to every new dataset (it was not generalizable); and the computational complexity required to run the algorithms obstructed its implementation in a Wireless Sensor Network (WSN) node, where real-time computing is a requisite.

To overcome these difficulties, an alternative methodology was explored in [24]. Up to nine standard algorithms (with no ad hoc tuning) were considered in a non-sequential frame-by-frame classification scheme. These classifiers did not take into account the order of the frames, and the final labelling of a sound was achieved by simply counting the number of frames belonging to each class. For comparison purposes, a pure sequential classifier, the Hidden Markov Model (HMM), was also considered. The experimental results show that the proposed method clearly outperforms the HMM, thereby demonstrating that the non-sequential classification of anuran sounds is feasible. From among the algorithms tested, the decision-tree classifier showed the best performance with an overall classification success rate of 87.30%, which is a particularly striking result considering that the analysed sounds were affected by a decidedly noisy background.

In an effort to exploit the information contained in the order of frames, six classification methods were proposed in [25], all of which were based in the data-mining domain. The comparison of these sequential classification methods revealed that they can obtain a slightly better performance than their non-sequential counterparts. The sliding window approach with an underlying decision tree attained the best results in the experiments: a noteworthy overall accuracy of 90.48%.

The implementation aspects in environmental monitoring systems were explored in [26], whereby the time required to compute every step in the classification process was considered: feature extraction; training classifier; and non-sequential and sequential classification. It was shown that it was feasible to operate many anuran sound classifiers in real time, particularly those obtaining the best classification performance.

### 1.3. Research Objectives

Based on this background, the main aim of this paper is to explore the feature extraction process, that is, to analyse the best way to represent the information contained in a sound frame. Further to the MPEG-7 features used in previous work, several other ways to represent every frame spectrum will be considered, from Filter Bank Energy (FBE) to the commonly used Mel Frequency Cepstral Coefficients (MFCC). However, the extraction of FBE and MFCC features is a complex process that has many options. In the paper, the optimal way of representing the spectrum of anuran calls will also be experimentally explored in order to attain the best classification results.

Additionally, a much more extensive dataset has been employed in the experiments, whereby more than 850 sound recordings of four different classes have been included, using up to 10 different classifiers and seven different performance metrics.

It will be shown in this paper that representing the frame spectrum with pure FBE offers slightly worse results than those obtained by employing the MPEG-7 features. Nevertheless, this performance can easily be increased by rescaling the FBE in a double dimension: vertically, by taking the logarithm of the energies; and horizontally, by applying a mel scaling in the filter banks. Moreover, selecting the proper values for the options in the feature extraction process will also provide further gain in several metrics. On the other hand, the representation of the spectrum in the cepstral domain, as in MFCC, has shown additional marginal improvements in classification performance. The overall result is that, by the optimal representation of the anuran call spectrum, its classification performance can be noticeably increased, and can obtain an accuracy that is ten points higher than the MPEG-7 counterpart.

## 2. Materials and Methods

### 2.1. WSN Architecture

The use of wireless sensor networks to monitor natural habitats has become a common methodology to ease the research tasks of biologists. The reasons for the many advantages in applying this technology to this area of knowledge lie in the features of the sensor nodes: capacity of measurement, data processing, computation, wireless communication, and energy autonomy. In addition, the design of such nodes focuses on minimizing the power consumption and the economic cost of the network. In this way, a long lifetime of the network and a large area of deployment can be achieved.

The architecture of our network is based on this philosophy of design. Two kinds of nodes have been considered: base station nodes and terminal nodes. There are usually only a few base station nodes and hundreds of terminal nodes.

The main goal of the base station nodes is to collect information from the whole network and to integrate it into an infrastructure network, such as Transmission Control Protocol-Internet Protocol (TCP-IP), Ethernet, General Packet Radio Service (GPRS) and Long-Term Evolution (LTE). In this respect, base station nodes behave as gateways between a wireless sensor network and an infrastructure network managed by a communication service provider. Consequently, base station nodes have two different network interfaces: one for the infrastructure network and the other for the wireless sensor network. Although the bandwidth of the infrastructure network could be high, the bandwidth used in the WSN interface is limited to the technology used. In our architecture design, several nodes are spread over a large area (hundreds of km^2^). Consequently, wireless communication has to consider long-range radio. To this end, we use two standard bands: the 2.4 GHz band, with greater bandwidth although with less penetration in the vegetation and, therefore, less range; and the 868 MHz band, which uses the free radio frequency spectrum. The data rate is limited in these bands to only a few kB/s.

From the point of view of power consumption, base station nodes can be considered as being located next to a communication cabinet, where the connection to the infrastructure network is implemented. In this cabinet, there used to be an external supply of electricity. According to this assumption, no autonomous electricity generation is needed for consideration in the design of base station nodes.

From the point of view of the computational capacity, base station nodes have to be able to deal with a huge amount of data that has been transmitted by remote nodes wirelessly. In order to process this data, base station nodes can run two types of algorithms: data aggregation and data fusion.

Data aggregation algorithms are focused on collecting data without considering the information that is being carried. The main goal of these algorithms is to minimize the data related with the protocol (overhead), thereby maximizing the payload.

The aim of data fusion algorithms is to minimize the size of the transmitted data, while focusing on preserving the meaning of the information that is to be delivered. In this respect, it is of utmost importance to correlate the information with the data. Data is the representation of the information. This interpretation of information allows us to minimize the size of the message that is to be delivered. Thus, while the data that is measured with a sensor could be based on an audio recording, the information that is to be exploited is the identification of presence of an individual of a specific anuran in this audio record. The reduction in data between sending the audio record and sending the information of the specific detected anuran is huge: from several kB to a mere dozen bytes. Moreover, in order to minimize power consumption, a sound threshold is established that activates the recognition system by generating an interruption in the microprocessor that launches a routine that addresses the acquisition of the audio and its processing. The node therefore only transmits information when a valid call is detected.

In this paper, the algorithms that are going to be described are considered from the point of view of the data fusion paradigm. However, they are implemented mainly in the terminal nodes. This strategy strives to reduce the data traffic in the wireless sensor network and to minimize the power consumption in communication tasks and minimizing the use of the electromagnetic band (the bandwidth is limited to only a few kB/s).

**Figure 1:**
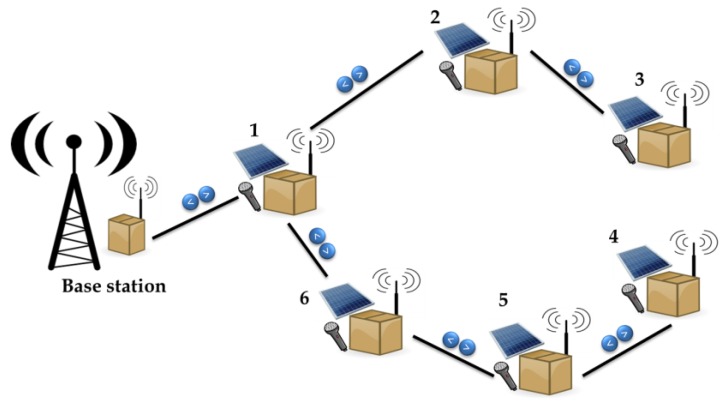
WSN architecture.

The terminal nodes have two main tasks: first, creation and maintenance of the wireless network in a collaborative way; second, collection of the information of its surroundings and its transmission to the base stations. The common way to create the wireless topology is based on a star. This way, it is easy to scale the network from dozens of nodes to hundreds. In this kind of network (spanning tree), the data is transmitted from the leaves to the root (Figure 1). One drawback has to be considered in such a network: as the number of nodes increases, the bottleneck effect at the root increases.

**Figure 2:**
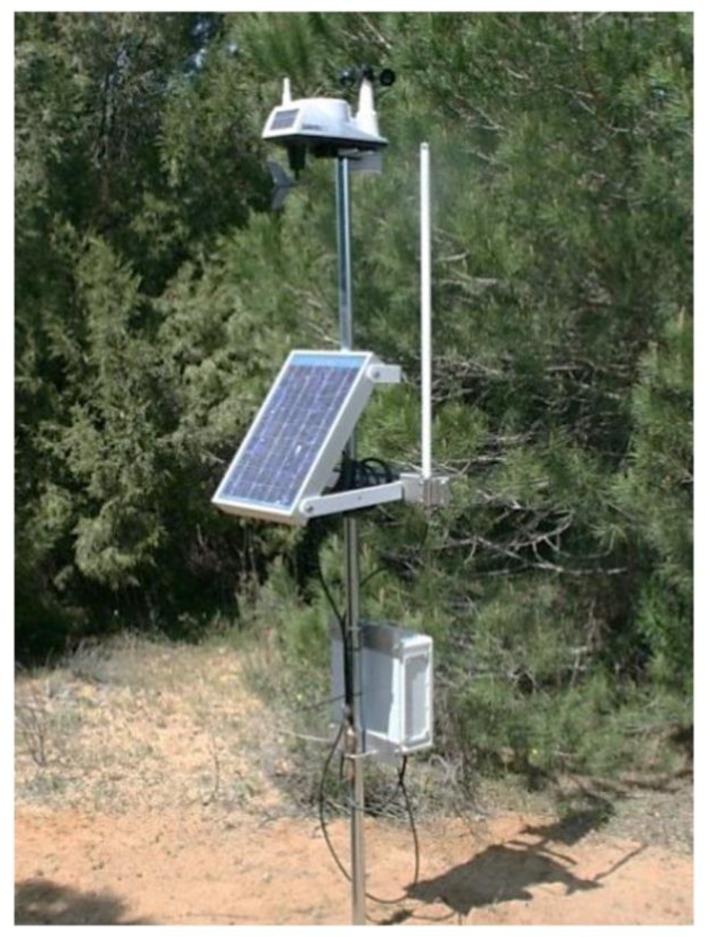
Typical terminal node.

The design of the terminal nodes considers an autonomous power supply (based on solar panels) and low power consumption (ARM microprocessors and low data rate transceivers). Furthermore, every node has an audio sensor for anuran identification and a set of meteorological sensors (temperature, humidity, etc.) for the description of the climate in which the identification is carried out. In Figure 2, a typical terminal node is shown [27].

### 2.2. Sounds Database

For testing purposes, actual anuran sounds provided by the National Natural History Museum (Museo Nacional de Ciencias Naturales) [28] have been employed (collection code starting on FZ0496). The sounds correspond to 2 species, the *epidalea calamita* (natterjack toad) and *alytes obstetricans* (common midwife toad), with a total of 868 recordings containing 4 classes of sounds:

1. *Epidalea calamita*; mating call (369 records),
2. *Epidalea calamita*; release call (63 records),
3. *Alytes obstetricans*; mating call (419 records),
4. *Alytes obstetricans*; distress call (17 records).

**Figure 3:**
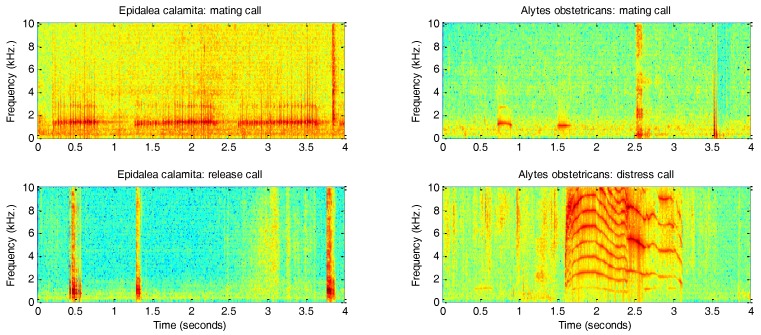
Spectrograms of sample calls for each sound class.

Figure 3 depicts the spectrograms of a sample call for each class. A total of 4343 s (1 h 13 min) of recordings has been analysed, with an average duration of 5 s.

**Figure 4:**
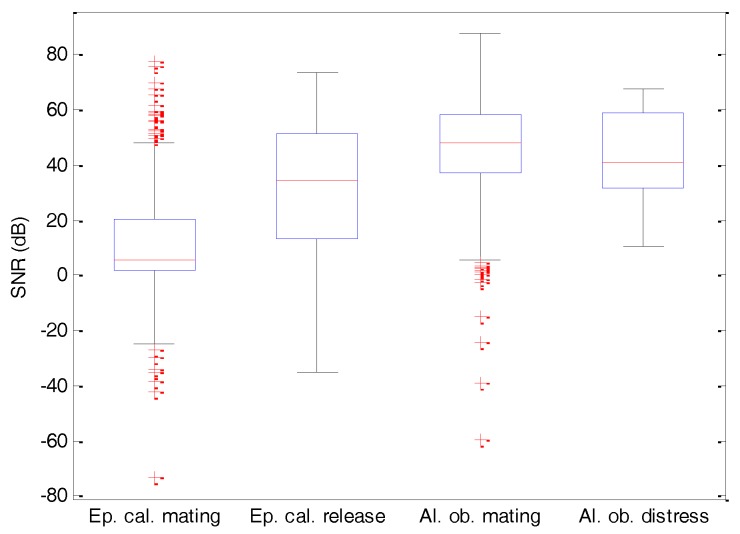
Signal-to-Noise Ratio (SNR) distribution for each sound class.

The sounds have been recorded in five different locations (four in Spain and one in Portugal) using a Sennheiser ME80 microphone (Wedemark, Germany), and this issue is discussed in detail in [23]. They are subsequently sampled at 44.1 kHz. A common feature of all the recordings is that they have been taken in their natural habitat, with very significant surrounding noise (wind, water, rain, traffic, voices, etc.), which posed an additional challenge in the classification process. The Signal-to-Noise Ratio (SNR) distribution for each sound class is depicted in Figure 4. The dataset presents an overall SNR median value of 35 dB, although some recordings have a much lower value.

In order to perform a supervised classification, certain sounds have to be selected as patterns (to be used in the training phase) while others are employed for testing. A common practice is to split the dataset into several disjoint subsets and apply a cross-validation technique. However, the use of these noisy recordings as patterns may lead to a decrease in the classification performance. Hence, several other approaches arise as an alternative to cross-validation. In our case, the recordings with relatively low background noise, which were carefully selected by biologists and sound engineers, have been used as patterns. This approach, usually called instance or example selection, is recommended in order to increase the rate of learning by focusing attention on informative examples [29,30,31,32].

To determine the frame patterns, the experts listen to the recordings of the anuran calls and simultaneously consider the spectrogram, and label each frame that they consider may belong to any of the possible classes. A total of 13 out of the 868 recordings have been selected as patterns with an SNR median value of 48 dB (13 dB higher than the full dataset). These recordings contain certain fragments of pattern sounds but also contain silence and/or noise sections. Table 1 summarizes the dataset of the sounds and patterns.

### 2.3. Sound Framing

**Figure 5:**
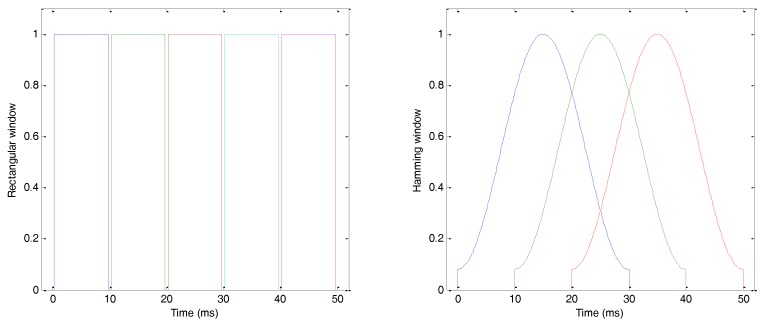
Sound framing in the time domain: (**left**) rectangular window function; (**right**) Hamming window function.

The first step to represent a sound is to split it up into frames of fixed duration. In the case of vocal sounds, this duration is usually related to the mechanism of production of sound and, specifically, to the period of opening and closing of the vocal cords, which is approximately 10 ms, both in humans [33] and in anurans [9]. By labelling s(n) as the discrete time-domain representation of the sound signal, a frame $s_{w}\left(n\right)$ is obtained using a window function w(n) in such a way that $s_{w}\left(n\right)=s\left(n\right)\cdot w\left(n\right)$. The simplest framing function is the rectangular window, which is 1 in the interval $\left[0,T_{w}\right]$, and 0 outside that interval. However, the framing process always introduces a distortion in the sound spectrum. In order to decrease this undesired effect, it is common to use a wider window of duration $T_{w}$ (for instance, 30 ms), to move the window forward in a shorter time $T_{s}$ (for instance 10 ms), and also use a bell-shaped window function. In this approach, each frame overlaps with the sides of the adjacent frames. One of the most commonly used window functions is the Hamming window, which is defined as

(1) $$w\left(n\right)=0.54+0.46\cos\left(\frac{2\pi n}{N}\right),$$

where N is the total number of values in the frame. Figure 5 represents a rectangular window function with $T_{w}=T_{s}=10\;\mathrm{ms}$ (left), and a Hamming window with $T_{w}=30\;\mathrm{ms}$ and $T_{s}=10\;\mathrm{ms}$ (right).

**Figure 6:**
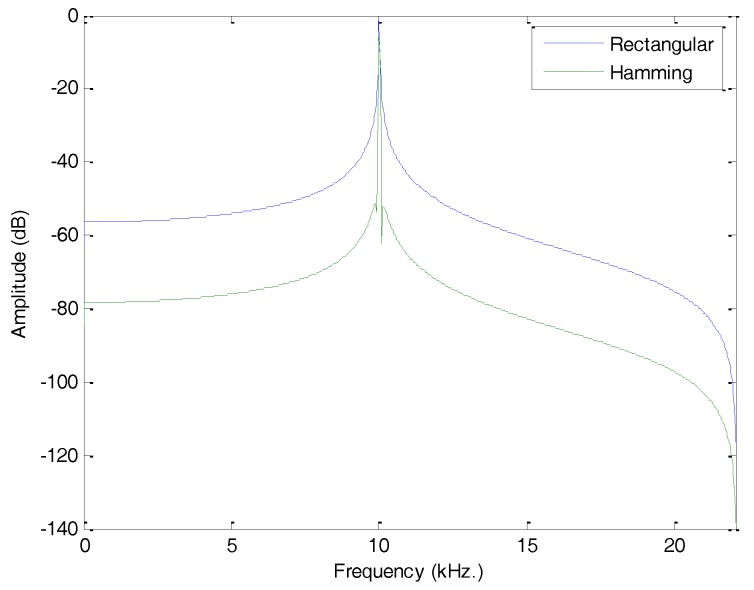
Effect of framing in the spectrum of a 10 kHz pure tone. Rectangular and Hamming window functions.

In order to show the effect of framing in the spectrum, a 10 kHz pure tone will be employed. Figure 6 depicts the spectrum obtained when using the simple rectangular function (blue) and the Hamming window (green). In both cases, the spectrum is altered by the framing process, but it can easily be seen that the Hamming window has significantly reduced the impact on the creation of undesired harmonics.

### 2.4. Spectrum Representation

**Figure 7:**
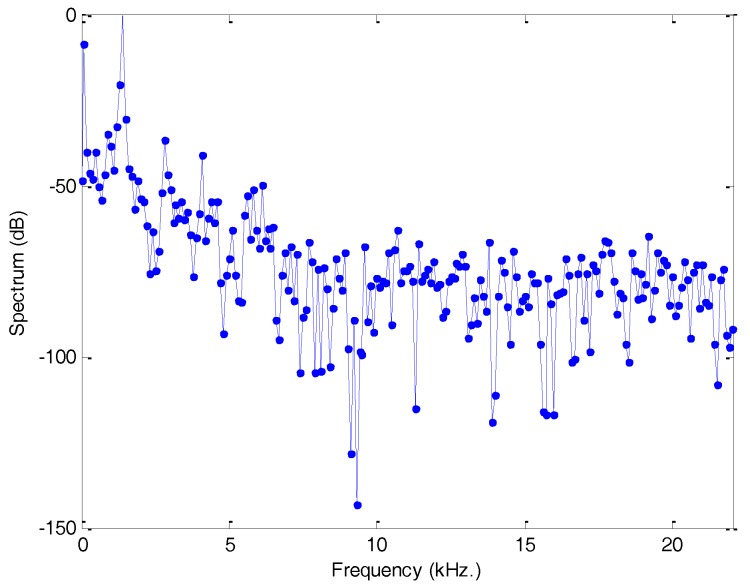
Typical spectrum of an anuran sound frame.

The representation of sounds is usually based on the frames obtained in the previous step. The procedure for obtaining a vector of values representing a frame is called feature extraction. Most of these algorithms are based on some kind of description of the frame spectrum. Figure 7 depicts a typical spectrum of an anuran sound frame.

One of the first issues that has to be addressed in featuring the spectrum is the range of frequencies that will be considered as relevant. A broad spectrum bandwidth needs more values to be characterized and, in many cases, includes noise that should be avoided. On the other hand, a spectrum that is too narrow may discard relevant frequency components.

If a frame contains N values, its spectrum, usually computed using the Fast Fourier Transform, also contains N values. For instance, a frame of a sound sampled at 44.1 kHz, with a duration $T_{w}=30\;\mathrm{ms}$, contains N=44.1·30=1323 values. This is a large number to be efficiently used for classification purposes. It is also a large number if these values have to be stored and/or transmitted in a WSN.

#### 2.4.1. MPEG-7 Feature Extraction

A first approach to significantly reducing the number of values that represent a spectrum is to use some kind of signature or fingerprint of the spectrum. The idea is not to store the spectral amplitude at every frequency, but to determine certain general characteristics of the shape of the spectrum. With these purposes in mind, the MPEG-7 ISO-standard [34] has been used for feature extraction. From this recommendation, the most significant parameters for classifying purposes have been selected. By executing three different processes on each frame, a set of 18 parameters is derived. These parameters can be derived from the following spectrum-related analyses:

1. Spectrogram analysis. By applying the Fast Fourier Transform (FFT) to the frame values, a spectral representation S(f) is obtained for each frame. The 5 parameters derived from this spectrum are:
  - Total power.
  - Relevant power, that is, the power in a certain frequency band.
  - Power centroid.
  - Spectral dispersion.
  - Spectrum flatness.
2. Linear prediction coding (LPC) analysis. From the sound values, s(n), a model of the sound source is estimated. This model uses a harmonic sound generator, a random sound generator, and a digital filter defined by its characteristic polynomial A(z). The roots of this polynomial are complex numbers $z_{i}$ which can be stated as $z_{i}=r_{i}e^{j\theta_{i}}$, and play a key role in this technique by determining the formants. Through LPC analysis, the spectrum envelope can be obtained and 11 parameters can also be derived such as:
  - Frequency of the formants (only the first three formants are considered).
  - Bandwidth of the formants (only the first three formants are considered).
  - Pitch.
  - Harmonic centroid.
  - Harmonic spectral deviation.
  - Harmonic spectral spread.
  - Harmonic spectral variation.
3. Harmonicity analysis. From the sound values, s(n), its autocorrelation function ρ(k) is obtained as this function is an indirect way of describing a spectrum. The two parameters derived from this analysis are:
  - Harmonicity ratio.
  - Upper limit of harmonicity.

A more detailed description of this frame feature extraction can be found in [23] and in the MPEG-7 standard [34].

#### 2.4.2. Filter Bank Energy

**Figure 8:**
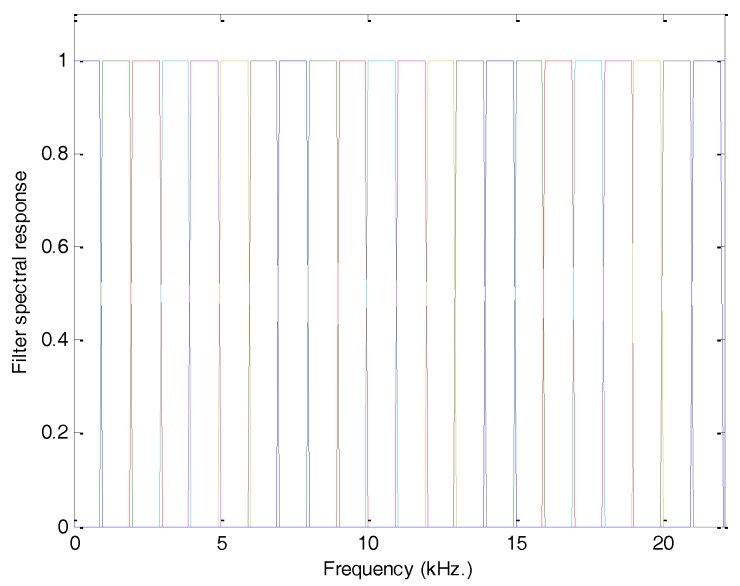
Bank of rectangular filters with a constant 1 kHz bandwidth.

A second approach to reducing the amount of information required to feature a spectrum is to compute the energy in a certain number of bands. To this end, a bank of M filters is used and the energy obtained for each filter is used to approximately describe the spectrum. Figure 8 depicts a bank of 22 rectangular filters, each with a constant 1 kHz Bandwidth.

**Figure 9:**
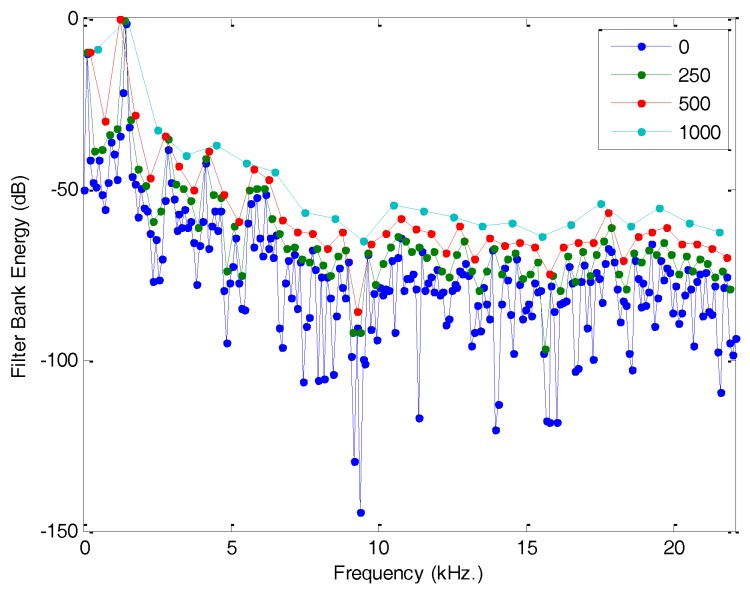
Filter Bank Energy (FBE) for a bank of rectangular filters with various bandwidths.

The result of applying a bank of filters to the original spectrum is called the Filter Bank Energy (FBE) and it is defined using only M values (usually a figure much smaller than N). Figure 9 reflects the FBE for a bank of rectangular filters with various bandwidths.

A widely used variation of the FBE is to apply an unevenly spaced bank of filters. In the field of sound classification, many studies are based on the hypothesis that automatic systems will obtain better results if they “imitate” human behaviour and, among other issues, take into account the different responses to signals of different frequency. It is a well-known fact about the human ear that:

- It has a lower sensitivity to low-frequency and, mainly, to high-frequency sounds [35]; and,
- It perceives two high-frequency tones as closer than a pair of equally spaced harmonics in the low-frequency range [36].

To reproduce this human-like behaviour, a scale of perceived tone is defined, called the mel (melody) scale, that arbitrarily assigns the value of 1000 mels at a frequency of 1 kHz and in which the constant increments of mel tones are perceived as evenly spaced by the human ear. This scale has been obtained experimentally and admits several formulations, the most popular of which is probably the following [37]:

(2) $$m=1197\ln\left(1+\frac{f}{700}\right).$$

**Figure 10:**
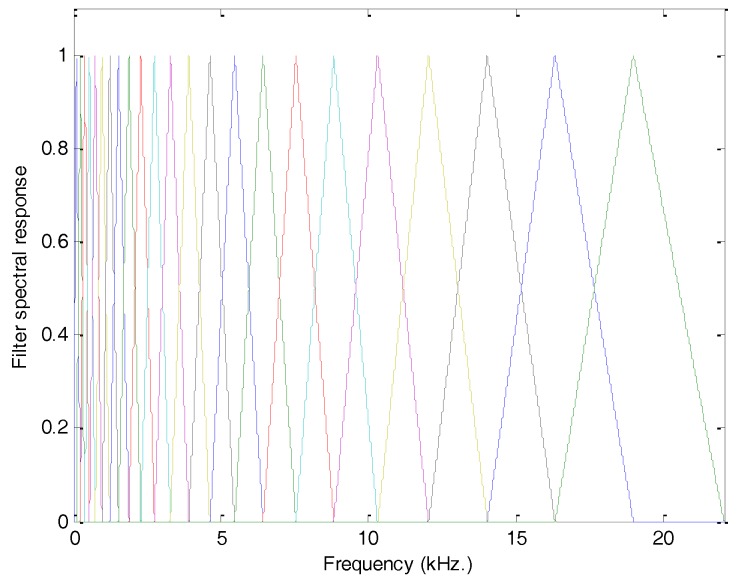
Spectral response of the 23 filter bank in conventional (frequency) scale.

By taking advantage of the mel scale, a bank of mel filters can be designed as one that is composed of filters whose spectral responses are isosceles triangles evenly spaced in the mel scale. In Figure 10, the spectral response of the mel filter bank in conventional scale (frequency) is presented (M=23).

**Figure 11:**
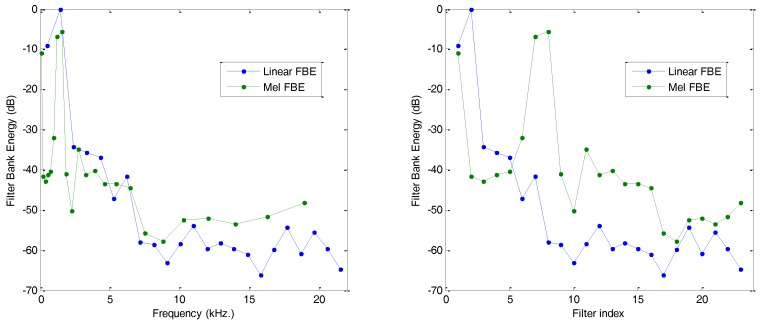
Linear and mel Filter Bank Energy: (**left**) horizontal frequency scale; (**right**) horizontal mel scale (filter index).

The effect produced by the application of this bank of filters is presented in Figure 11, where it is compared to the result obtained by rectangular filter banks of constant bandwidth (M=23). The effect on the Filter Bank Energy, as can be observed in the frequency scale (left), resembles some kind of equalization with a reduction in the low frequencies and an increase in the high frequencies. However, when the FBE is drawn in comparison to the filter index, then the most noticeable effect (right) is the horizontal rescaling of the spectrum with an expansion in the low frequencies and a compression in the high frequencies.

By applying the mel scale to the FBE spectrum, a certain improvement in classification performance should be expected.

#### 2.4.3. Cepstral Representation

A third approach for representing and compressing the spectrum information of a sound frame considers the Filter Bank Energy (FBE) as a periodical signal that can be expanded using a certain form of Fourier trigonometric or exponential series. However, since FBE is a spectrum, its Fourier expansion constitutes the spectrum of a spectrum, which is known as the cepstrum.

**Figure 12:**
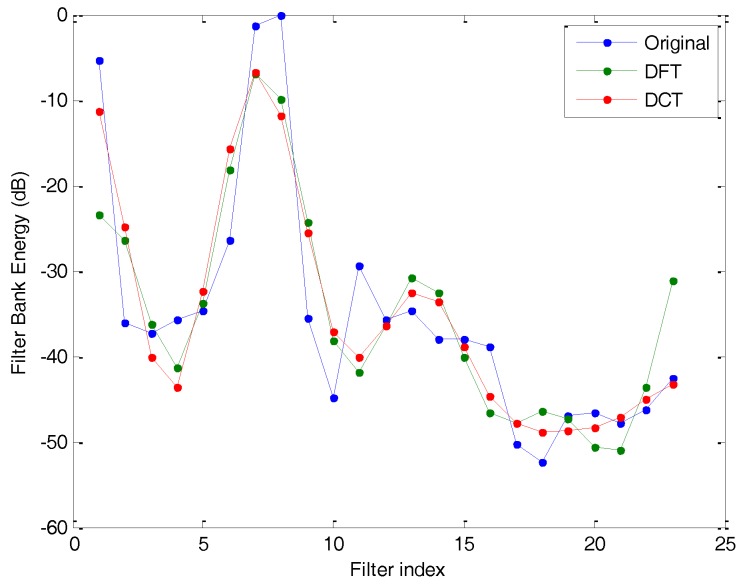
Original FBE of a frame and its approximate value using C = 10 harmonics (cepstral) components of the DFT and the DCT expansion.

The straightforward Fourier expansion is the Discrete Fourier Transform (DFT) or its faster Fast Fourier Transform (FFT) version. However, careful consideration of the FBE shows that, just as for every spectrum, FBE shows an even symmetry and, therefore, the Discrete Cosine Transform (DCT) would better suit this case. Figure 12 depicts the original FBE of a frame and its approximate value using C=10 harmonics (cepstral) components of both the DFT and the DCT expansion.

**Figure 13:**
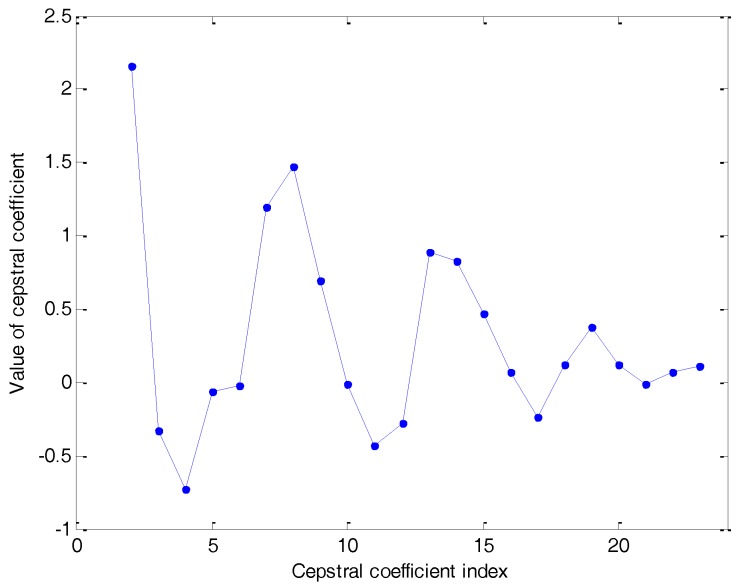
The DCT cepstral representation of an anuran sound frame.

Although both the cepstral representations offer similar results, a more detailed analysis should show that DCT has a lower error representing the FBE. Additionally, it is usual that the C coefficients obtained through the DCT have a lower cross-correlation than their DFT counterpart and, moreover, fewer cross-correlated coefficients should indicate better classification results. The DCT cepstral representation of the anuran sound frame used as the example is drawn in Figure 13.

**Figure 14:**
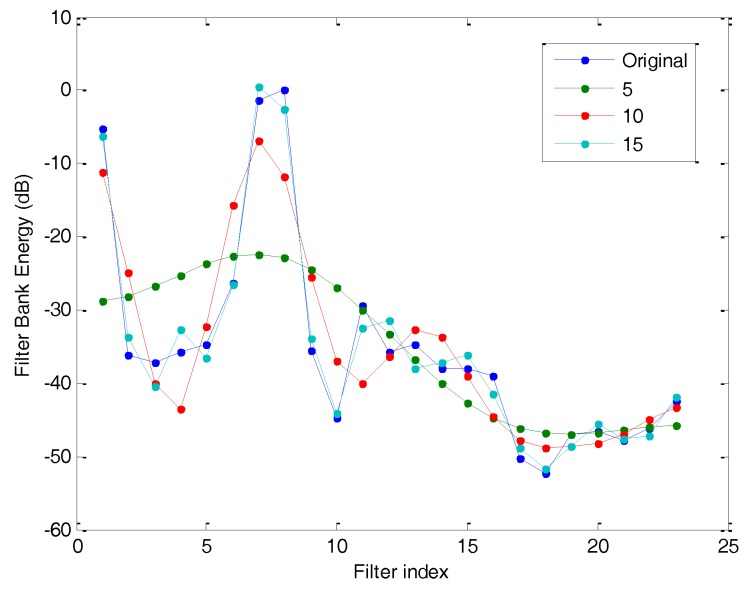
Original and approximate value of the FBE for different numbers of cepstral coefficients.

In order to reduce the size of the vector representing the frame spectrum, low values of *C* are desirable. In Figure 14, the approximate value of the FBE for different numbers of cepstral coefficients is depicted. In this example, despite reducing the spectrum representation from M=23 to C=10 values, they continue offering a very good approximation of the spectrum.

#### 2.4.4. Sound Pre-Emphasis

Vocal sound signals generally have less energy in the high-frequency band than in the low-frequency band. However, noise has a frequency behaviour of a more uniform nature to such an extent that, in many cases, it is usually modelled as white noise, that is, noise with a flat spectrum, which means that it has the same energy in any frequency band.

**Figure 15:**
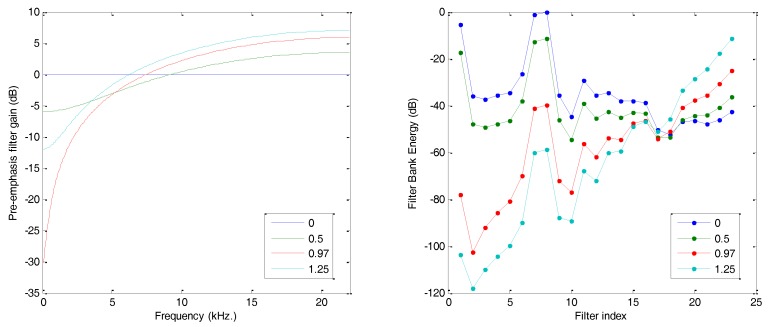
Pre-emphasis filter: (**left**) filter spectral response; (**right**) effect on FBE.

The combination of the two previous circumstances means that the signal-to-noise ratio (SNR) is, in general, significantly lower at high than at low frequency. This disparity in the value of the SNR can cause the influence of the high-frequency components in the classification processes to be greatly diminished. To correct this circumstance, it is usual to pre-filter the sound signal before representing its spectrum, which increases the relative importance of the high frequencies versus the low frequencies. To this end, a first-order digital filter is usually employed, which is given as:

(3) s′(n)=s(n)−α s(n−1),

where s(n) is the value of the sound sample at the n-th instant and α is a constant. Figure 15 (left) shows the spectral response of such a filter, called the pre-emphasis filter, and its effect on the Filter Bank Energy example (right).

#### 2.4.5. Cepstral Liftering

The cepstral coefficients obtained with the procedure described in the previous sections have a problem: the values of the higher-order coefficients are numerically small and this causes a very wide range of variances among the low-quefrency and high-quefrency cepstral coefficients. For pragmatic reasons, such as plotting the parameters of the model, it is convenient in certain cases to scale the cepstral coefficients to have similar magnitudes.

**Figure 16:**
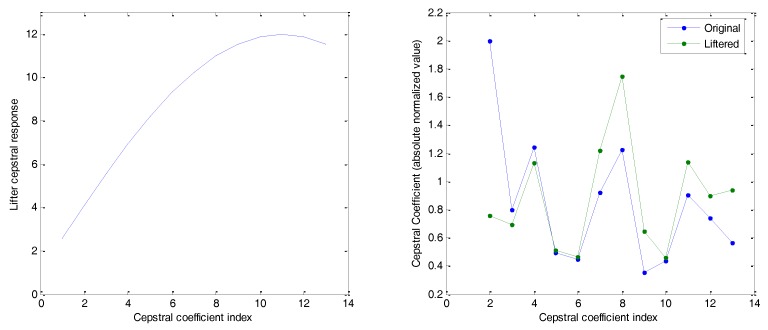
Cepstral sine lifter: (**left**) lifter cepstral response; (**right**) effect on cepstrum.

For this reason, certain implementations include a final processing in the calculation of the coefficients that increases the relative value of the high-quefrency coefficients. To this end, a lifter (a filter in the cepstral domain) is used, which is given by the following expression:

(4) $$c_{ik}^{\prime }=\left(1+\frac{L}{2}\sin\frac{\pi i}{L}\right)c_{ik},$$

where $c_{ik}$ is the i-th cepstral coefficient of the k-th frame, $c_{ik}^{\prime }$ is the same coefficient after the liftering process, and L is a parameter of the lifter. Figure 16 (left) depicts the cepstral response of such a lifter (L=22), and its effect on the example Cepstral Coefficients (right). It shows the absolute values of the cepstral coefficients, once normalized for ease of comparison. The relative increase in the high-quefrency cepstral coefficients can be observed.

#### 2.4.6. Mel Frequency Cepstral Coefficients (MFCCs)

If the sound spectrum is rescaled considering the mel scale and it is later represented using the cepstral coefficients, then the resulting feature vector is denominated Mel Frequency Cepstral Coefficients (MFCCs). In this case, no standard set of options has been universally adopted. However, an European Telecommunications Standards Institute (ETSI) standard covers certain applications in the mobile telephone realm [38], and a widespread implementation originally developed by Cambridge University, the Hidden Markov Model Toolkit (HTK) [39], recommends a number of by-default options.

**Figure 17:**
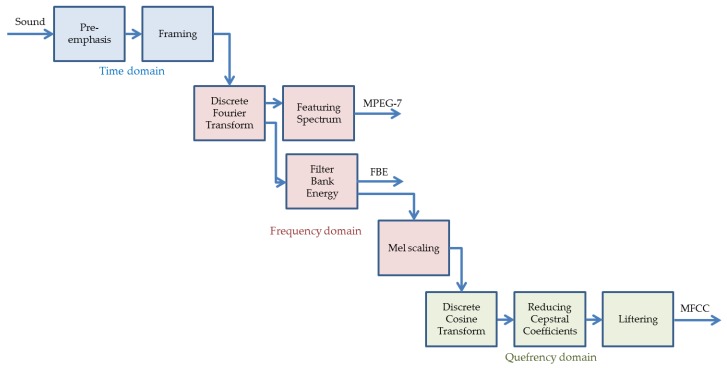
Overall structure of the process for the representation of a spectrum.

Figure 17 reflects the full process of representing a spectrum, by showing every process in the three domains (time, frequency, and quefrency), and by depicting the 3 alternatives to represent an anuran call spectrum: MPEG-7 spectrum features, Filter Bank Energy, and MFCC cepstral representation.

### 2.5. Sound Classifiers

To tackle the classification process, the sound dataset has to be split into 3 subsets. Firstly, recordings with relatively low background noise, which were carefully selected by biologists and sound engineers, have been used as patterns. In this research the training dataset contains 13 records. The parameters for each classifier are determined by exclusively using these pattern records. The remaining elements in the dataset are then randomly divided into two approximately equal subsets used for validation and testing. The validation dataset, containing 430 records, is employed to determine the hyper-parameters of the classifiers. On the other hand, the testing dataset containing 425 elements, which includes none of the patterns or validation sounds, is employed for the evaluation of the performance of each algorithm. Table 1 summarizes the dataset of the sounds and patterns.

By means of the feature extraction procedures described in the previous subsection, each sound frame (its spectrum) is characterized by D parameters or, equivalently, by a point in an ${\mathbb{R}}^{D}$ space defined by its coordinate vector $s=\left[s_{1},s_{2},\ldots ,s_{D}\right]$. N pattern frames are also available where the i-th pattern is additionally represented by a point in the ${\mathbb{R}}^{D}$ space with a coordinate vector $x_{i}=\left[x_{i1},x_{i2},\ldots ,x_{iD}\right]$. Each frame is labelled as belonging to a certain class θ out of a total of M classes. The set of pattern frames can be seen as a cloud of points in ${\mathbb{R}}^{D}$ and can be identified by a matrix $\Pi =\left[x_{1},x_{2},\ldots ,x_{N}\right]\prime$ containing the coordinate vector of the N points. The subset of points in Π belonging to the class θ is denoted by its matrix $\Pi_{\theta }$. Non-sequential classifiers perform a certain type of comparison between the frame to be classified (represented by its vector s) and the pattern frames (represented by its matrix Π). This comparison is carried out in the space of the ${\mathbb{R}}^{D}$ features and its result is called a supervised classification.

A wide and representative set of non-sequential supervised classifiers has been considered. Additionally, the sequential supervised HMM classifiers have been examined. The set of ten classification procedures used in this paper include: Minimum distance (MinDis) [40], Maximum likelihood (MaxLik) [41], Decision trees (DecTr) [42], k-nearest neighbours (kNN) [43], Support vector machine (SVM) [44], Logistic regression (LogReg) [45], Neural networks (Neur) [46], Discriminant function (Discr) [47], Bayesian classifiers (Bayes) [48], and Hidden Markov Models (HMM) [49] .

Although the concluding results have to be implemented in the WSN nodes, a previous desktop prototype has been designed to perform the comparisons in the feature extraction process and in the classification algorithms. For this reason, the ten aforementioned classifiers have been prototyped using MATLAB (2014a, Mathworks, Natick, MA, USA). The minimum distance classifier in its training phase obtains the mean value $\mu_{jk}$ for the j-th feature belonging to the k-th class. In the test phase for every frame, the distance $d_{k}$ between the frame features and the mean value of the k-th class is obtained in accordance with the expression:

(5) $$d_{k}=\sqrt{\sum_{j=1}^{D}{\left(x_{j}-\mu_{jk}\right)}^{2}},$$

where $x_{j}$ is the value of the j-th feature. The class assigned to the frame is that with the minimum distance.

The maximum likelihood classifier is used under a Gaussian probability distribution with full covariance. The neural network classifier is based on a feed-forward neural network with a 10-neuron hidden layer and a 1-neuron output layer. The remaining methods and classifiers have been coded based on built-in MATLAB functions using their default parameters, which are reflected in Table 2. A more detailed description of the classifiers employed can be found in [24,50].

### 2.6. Classification Metrics

The definition of the most suitable classification performance metrics represents a key aspect in the evaluation of procedures, and it is difficult to overstate its importance [51]. In order to compare the results obtained for every classifier and every combination set of features, several metrics for the performance can be defined [52], all of which are based on the binary confusion matrix (see Table 3).

The most relevant metrics and their definitions are shown in Table 4, where they are computed for each class (considered “positive”), thereby leaving the remaining classes to be called “negative”. Additionally, an average per class can be defined for each metric.

Since the number of instances in every class remains imbalanced in our dataset (see Table 1), the use of accuracy or precision as the main performance metric can imply a significant skew [54]. It is therefore preferable to use sensitivity and specificity since they are unbiased metrics even when the classes are imbalanced. Therefore, when a single metric is required to compare classifier results (i.e., to identify “the best classifier”), the Receiver operating characteristic (ROC) values and the Geometric Mean are preferred as they combine, in a single metric, the sensitivity and the specificity, which both present a better behaviour in the presence of imbalanced classes [55].

## 3. Results

In this section, we present the results obtained in a set of experiments conducted to obtain the optimal representation of the anuran call spectrum in order to provide a more efficient classification. To this end, the dataset described in Section 2 has been employed and the spectrum of every sound frame has been featured using several approaches. The features extracted were then used to classify the sounds using the classifiers also described in the previous section.

For spectrum representation, three alternatives were selected: the MPEG-7 features, the Filter Bank Energy, and the MFCC features using the HTK default options. Any of these alternatives uses the set of options that are summarized in Table 5.

The classification performances have been obtained for each of these cases. Additionally, the impact on the classification performance of every option in the MFCC extracting procedure has been explored, which enables an optimal set of options to be selected during their extraction. This optimum set of values is also presented in the last column of Table 5.

### 3.1. Sound Classification Using MPEG-7 Features

**Figure 18:**
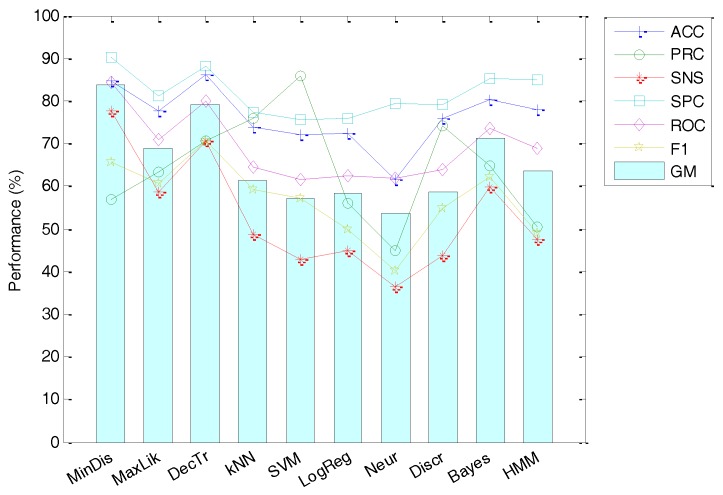
Classification performance using MPEG-7 features.

MPEG-7 feature extraction has the advantage of its standardization, and hence no optional parameters have to be adjusted and selected. Therefore, using the recommended values, the classification performance can be summarized in Figure 18. It can be seen that the best result is obtained by the Minimum Distance classifier with an accuracy of 85%.

### 3.2. Sound Classification Using Filter Bank Energies

**Figure 19:**
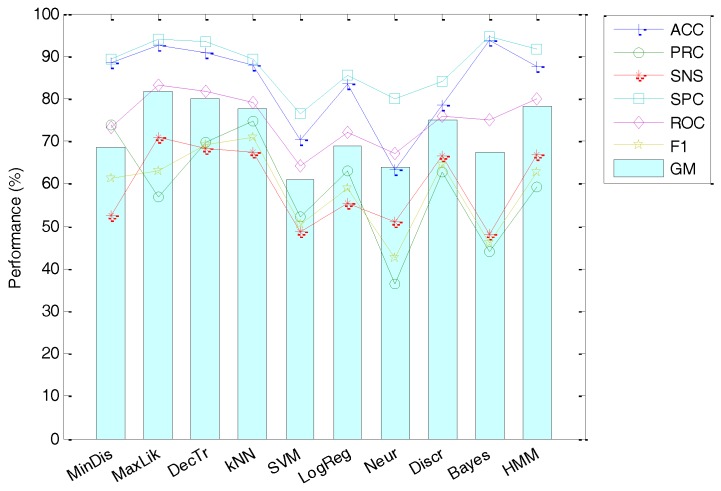
Classification performance using Filter Bank Energy.

The second approach represents the anuran call spectrum using the Filter Bank Energy. As there is no standard or common practice in selecting options for FBE, we have used the same options as in MPEG-7 to facilitate comparison. The classification performance obtained using this strategy can be summarized in Figure 19. It can be observed that the best results are attained using the Maximum Likelihood classifier with an accuracy of 92.69%.

### 3.3. Sound Classification Using MFCC (Default Options)

**Figure 20:**
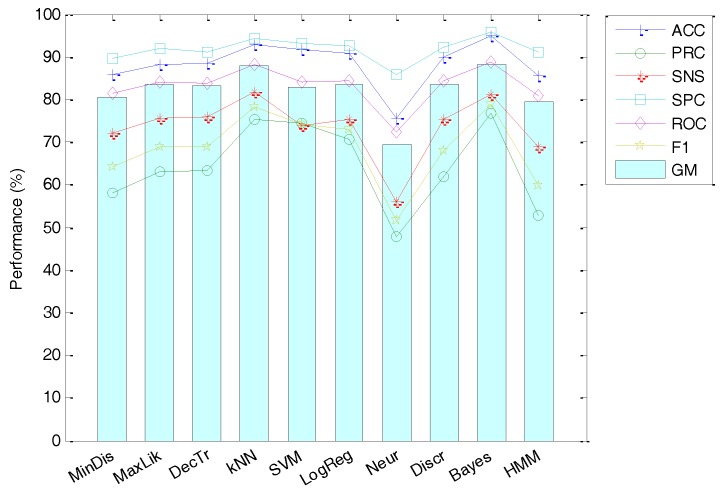
Classification performance using MFCCs (default options in the HTK implementation).

The third alternative involves the use of the MFCCs as the vector featuring an anuran call spectrum. As a starting point, the default values used in the MFCC-HTK implementation (see Table 5) are used for every option. The classification performance obtained using this strategy can be summarized in Figure 20. It can be seen that the best results are obtained using the Bayes classifier with an accuracy of 94.85%.

### 3.4. Classification Performances versus MFCC Feature Extraction Options

From among the three alternatives explored in the previous subsections, featuring the anuran call spectrum using MFCC has resulted in the best classification performances. However, the process of extracting these MFCC features leaves plenty of options as summarized in Table 5. It is now time to investigate whether a different set of values for the extracting options could achieve even better classification results.

The straightforward mechanism to find the optimum values for the options should include an exhaustive search in the option space, which has dimension 11 (see Table 5). Considering that the number of values for each option is $O_{1},O_{2},\cdots ,O_{11},$ the total amount of combinations to be explored will be $O_{1}\cdot O_{2}\cdots O_{11}$ which is usually a very large number. For instance, on considering 10 values for each option, then the number of combinations would be $10^{11}$. Furthermore, for each combination of option values, the full sound dataset (868 recordings) has to be catalogued using the 10 classifiers, and then its performance obtained. Since the evaluation of every point in the option space takes about 30 min on a desktop computer, it would therefore be unfeasible for practical reasons to carry out a full search.

Alternatively, a much simpler and faster but still effective approach has been employed. We consider a starting point in the option space and each time we move in a single dimension. The first search will consider the starting point (1) and the remaining $O_{1}-1$ values of the first option. Searching in the second dimension will need the computation of $O_{2}-1$ values. The number of evaluations will therefore be:

(6) $$1+\left(O_{1}-1\right)+\left(O_{2}-1\right)+\cdots +\left(O_{11}-1\right)=O_{1}+O_{2}+\cdots +O_{11}-10.$$

Considering again 10 values for each option, the number of combinations will be 100, a much more affordable search. As the starting point, the default values of the MFCC-HTK implementation have been used.

**Figure 21:**
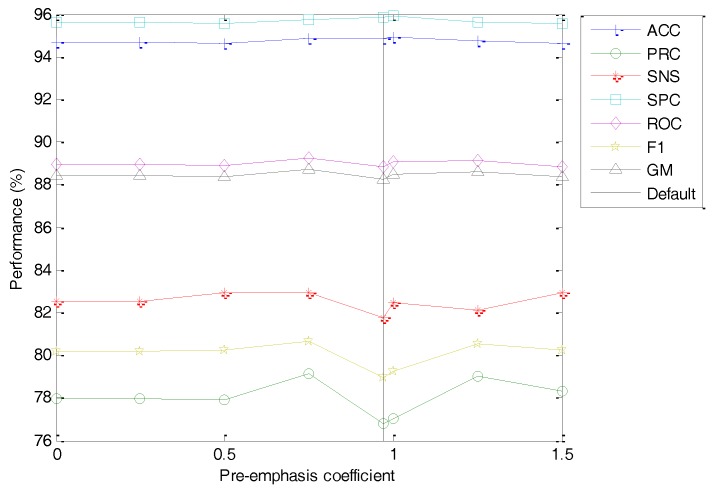
Classification performance metrics of the best classifier vs. the value of the pre-emphasis coefficient.

For the search for every option, the order described in Table 5 is followed, and hence the first option to be considered is the pre-emphasis coefficient. The classification performance metrics of the best classifier vs. the value of this coefficient is depicted in Figure 21 (with the dashed line indicating the default value). No concluding value arises from this study (an almost flat dependency), and, hence, this coefficient apparently has no influence on the overall performance. For a faster extraction, eliminating the pre-emphasis stage is suggested.

Let us now consider the options related to the framing process by first exploring the influence of the window function. The simplest rectangular window is compared to the more advanced Hamming (default) function. The performance metrics for the best classifier are reflected in Table 6. It can be observed that using the Hamming window function significantly increases classification performance.

**Figure 22:**
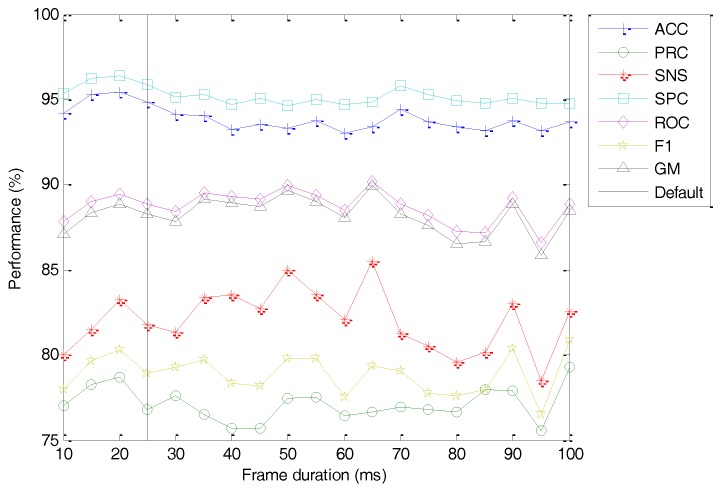
Classification performance metrics of the best classifier vs. the frame duration.

Still regarding the framing process, the second option to be considered is that of the frame duration ($T_{w}$). The classification performance metrics of the best classifier vs. the value of this option is depicted in Figure 22 (with the dashed line indicating the default value). As can be observed, a smaller value of the frame duration ($T_{w}=20$) slightly increases the classifier accuracy.

**Figure 23:**
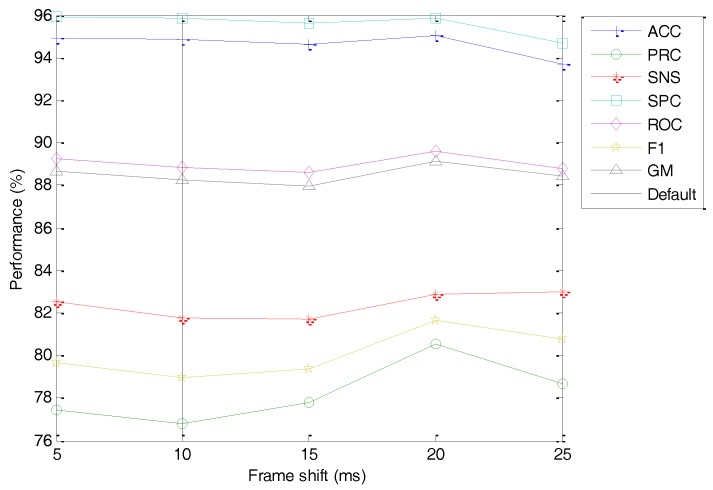
Classification performance metrics of the best classifier vs. the frame shift.

The last analysis of the framing process takes into account the frame shift ($T_{s}$). The classification performance metrics of the best classifier vs. the value of this option is depicted in Figure 23 (with the dashed line indicating the default value). No concluding value arises from this study (an almost flat dependency), and hence it appears that this coefficient has a very limited influence on the overall performance. Therefore, the $T_{s}=10$ default value is maintained.

**Figure 24:**
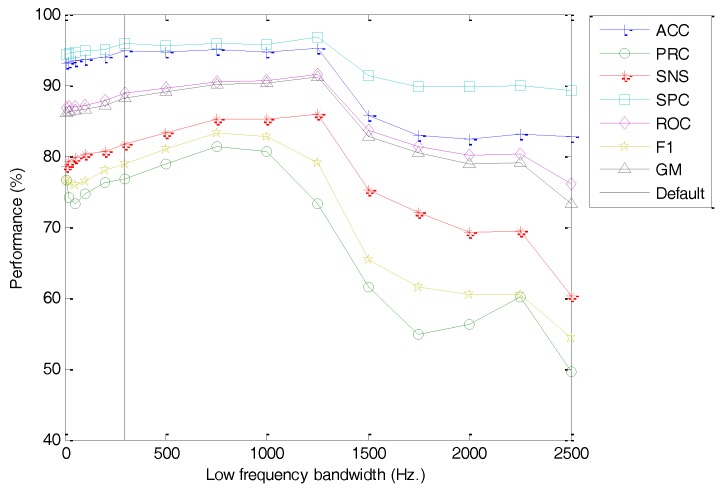
Classification performance metrics of the best classifier vs. the low-frequency limit.

Moving forward, the options involved in the process of obtaining the Filter Bank Energy are now analysed. Its first element should be the low-frequency limit of the spectrum ($L_{f}$). The classification performance metrics of the best classifier vs. the value of this option is depicted in Figure 24 (with the dashed line indicating the default value). For small values of the low-frequency limit, an increase leads to better performance, probably due to a limitation on the influence of the low-frequency noise. On exceeding a certain threshold (of about $L_{f}\geq 1000\;\mathrm{Hz}$), however, the performance decreases, probably because relevant harmonics are discarded below this frequency.

**Figure 25:**
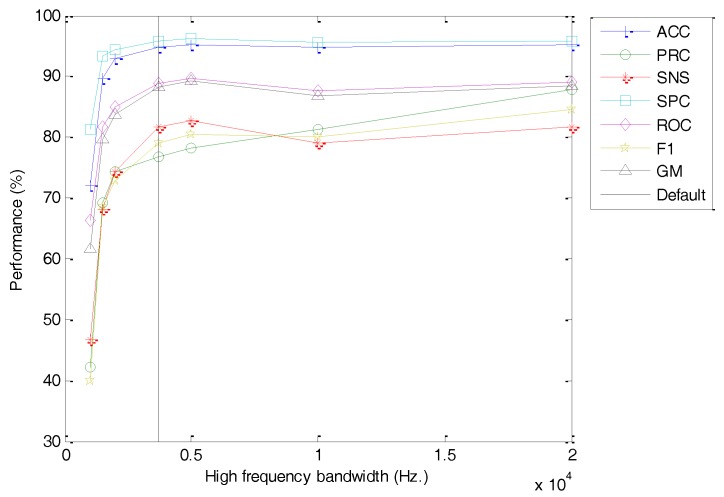
Classification performance metrics of the best classifier vs. the high-frequency limit.

An analogous study has been carried out on the high-frequency limit of the spectrum ($H_{f}$). The classification performance metrics of the best classifier vs. the value of this option is depicted in Figure 25 (with the dashed line indicating the default value). For large values of the high-frequency limit, an almost flat response is obtained. On exceeding a certain threshold (of about $H_{f}\leq 5000\;\mathrm{Hz}.$), however, the performance decreases, probably because relevant harmonics are discarded above this frequency. The optimum values for the spectrum bandwidth should maintain the highest possible values for performance metrics, but with the most limited frequency range in order not to increase the number of filter banks required (and later the size of the spectrum feature vector).

**Figure 26:**
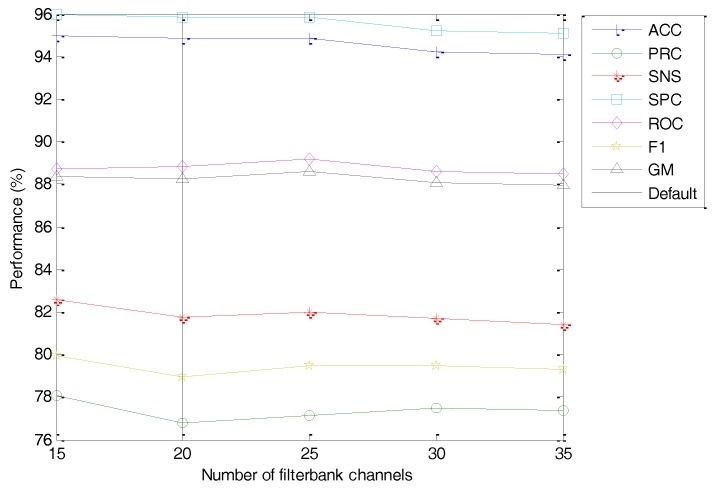
Classification performance metrics of the best classifier vs. the number of filter banks.

Having considered the frequency range, it is time to focus on the number of filter banks (M). The classification performance metrics of the best classifier vs. the value of this option is depicted in Figure 26 (with the dashed line indicating the default value). No concluding value arises from this study (an almost flat dependency) and hence it appears that this coefficient has a very limited influence on the overall performance. This is probably due to the fact that changing the value of M while maintaining a smaller value of the number of cepstral coefficients (C=13) has a very limited influence. Therefore, the M=20 default value is maintained.

The last analysis of the Filter Bank Energy process will take into account the scaling of the frequency axis. The simplest rectangular filter bank will be compared to the mel filter bank. The performance metrics for the best classifier are reflected in Table 7. It can be seen that using the mel scale slightly increases classification performance.

Moving now to the analysis in the quefrency domain, the options involved in the process of obtaining the Cepstral Coefficients are analysed. Its first element should be the type of transform to obtain the cepstrum. The straightforward DFT is compared to the DCT, which takes into account the even symmetry of the Filter Bank Energy. The performance metrics for the best classifier are reflected in Table 8. It can be observed that both transforms offer very similar results with a slight advantage for the DCT (the default option).

**Figure 27:**
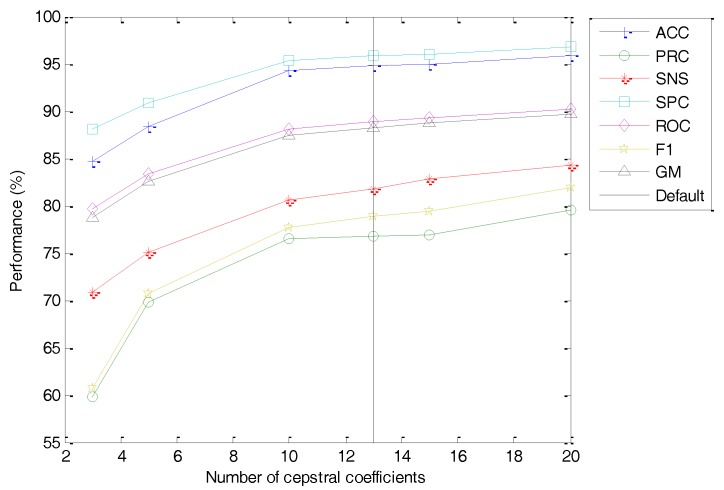
Classification performance metrics of the best classifier vs. the number of cepstral coefficients.

In the quefrency domain, probably the most relevant option should be the number of cepstral coefficients (C) approximating the Filter Bank Energy. The classification performance metrics of the best classifier vs. the value of this option is depicted in Figure 27 (with the dashed line indicating the default value). As shown, a smaller value of the number of cepstral coefficients (C) worsens the classifier performance metrics. However, since this effect is limited for a small reduction of C, by using half the number of cepstral coefficients C=10 (instead of the maximum C=20 when M=20) leads to only a slight reduction in performance (from ACC=95.85% to ACC=94.27%).

**Figure 28:**
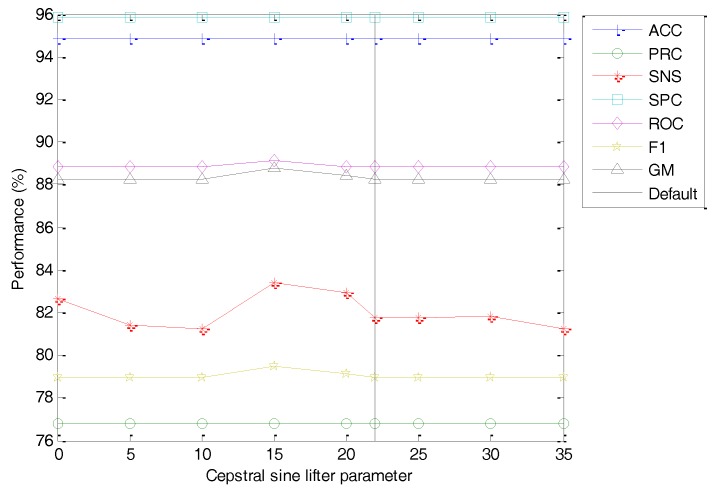
Classification performance metrics of the best classifier vs. the value of the cepstral lifter parameter.

Finally, the influence of the options in the liftering process is explored. The classification performance metrics of the best classifier vs. the value of this option is depicted in Figure 28 (with the dashed line indicating the default value). No concluding value arises from this study (an almost flat dependency), and hence it appears that this coefficient has no influence on the overall performance. For a faster extraction, the elimination of the liftering stage is suggested.

### 3.5. Sound Classification Using Optimal MFCC

**Figure 29:**
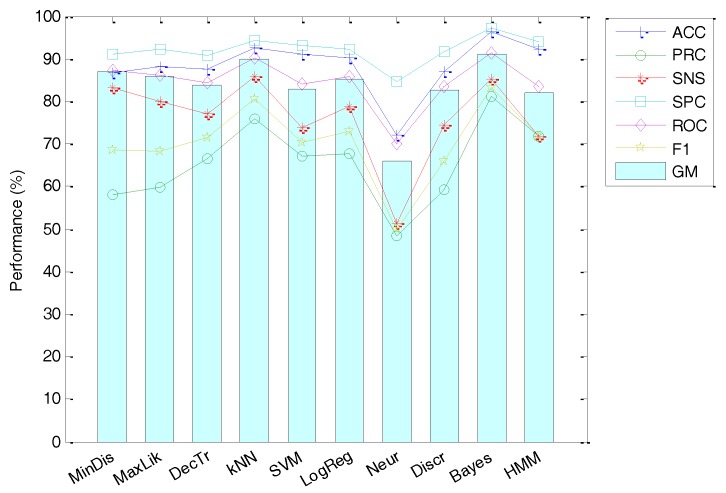
Classification performance using MFCCs (optimal values in every option).

Having investigated the effect of every option for the extraction of MFCC parameters in the classification performance metrics, an optimum set of values can be selected. Their values are indicated in the last column of Table 5. The classification performance attained using this strategy can be summarized in Figure 29. It can be seen that the best results are achieved using the Bayes classifier, with an accuracy of 96.37%.

Table 9 shows the confusion matrix obtained using the Bayes Classifier on MFCC features that have been extracted with the optimal values in every option. It can be seen that every class is well classified except the *epidalea calamita* release call. This is probably due to the fact that this sound is very short and has an almost flat spectrum, which makes it difficult, even for human experts, to distinguish it from a wideband spike noise.

## 4. Discussion

### 4.1. Comparing Classification Performances

**Figure 30:**
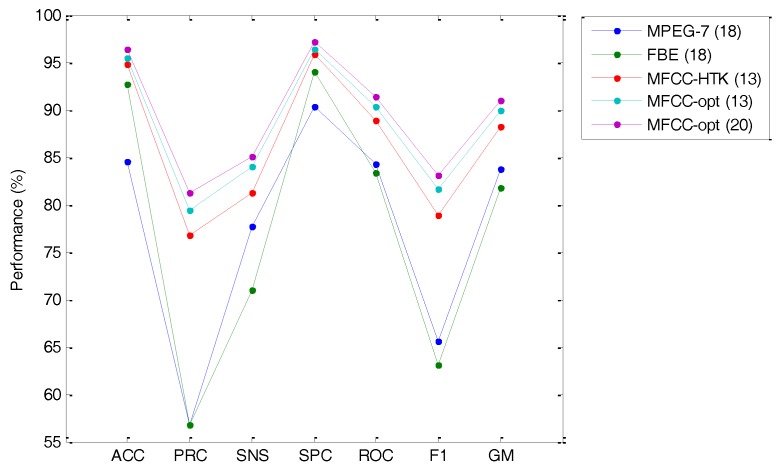
Classification performance using various alternatives for the representation of the anuran call spectrum.

In Section 3 above, basic alternatives for the representation of the anuran call spectrum have been explored: MPEG-7 18-feature set, 18-Filter Bank Energy, and 13-MFCC following the HTK default implementation. Additionally, we have made an extensive search for the optimum values of the MFCC feature extraction and an optimal set of values for these options has been selected. Table 10 and Figure 30 summarize the classification performance metric for the three basic alternatives and for the optimized representation, using 13 and 20 features.

Regarding these results, it can be concluded that the cepstral representation of the anuran call spectrum offers the best performance from among the set of alternatives explored. The FBE approach can increase the accuracy over the MPEG-7 (but not over MFCC), although it does incur a noticeable decrease in other metrics, such as precision and sensitivity.

It can also be concluded that exploring the MFCC option space can slightly increase every performance metric (a value between 0.5% and 3% with the same number of features).

Moreover, extracting MFCC is much more efficient in terms of computing requirements than obtaining MPEG-7 features [26]. They are therefore much more convenient for implementation on real-time low-priced nodes.

### 4.2. Breaking Down the Improvement in Classification Performances.

In the previous section, it has been demonstrated that the representation of the spectrum with the Filter Bank Energy offers a similar classification result to that from using the MPEG-7 feature, although the use of MFCC clearly improves the classification performance. However, one question arises: What is the contribution towards the improvement of the performance of each stage on the way from FBE to MFCC?

In order to answer that question, the sounds in the dataset have been featured using the same number of parameters (18) and several extraction techniques (8) corresponding to each stage towards increasing performance:

1. MPEG-7 features (extracted with the options described in Table 5).
2. FBE (extracted with the options described in Table 5).
3. FBE in log-scale, that is, extracted with the same options used in the previous stage but using a logarithmic scale to represent the energies.
4. FBE in mel-log-scale, that is, extracted with the same options used in the previous stage but using a mel scale to represent the frequencies. In fact a mel filter bank, as described in Section 2.4.2, was used.
5. FBE in mel-log-scale with optimum options, that is, extracted with the same options used in the previous stage but using the optimum values for the remaining extracting options.
6. DCT (Discrete Cosine Transform) of the FBE in mel-log-scale, that is, the DCT of stage 4. This result is in fact a set of Mel Frequency Cepstral Coefficients (MFCC) but obtained with options that are not the default options defined in HTK, nor the optimum values obtained in Section 3.
7. MFCC with optimum frame duration ($T_{w}=20\;\mathrm{ms})$, that is, extracted with the same options used in the previous stage but using the optimum frame duration.
8. MFCC with optimum options, that is, extracted with the same options used in the previous stage but now using the optimum values for the limits of low frequency ($L_{f}=1000\;\mathrm{Hz}$) and high frequency ($H_{f}=5000\;\mathrm{Hz}$) of the spectrum.

**Figure 31:**
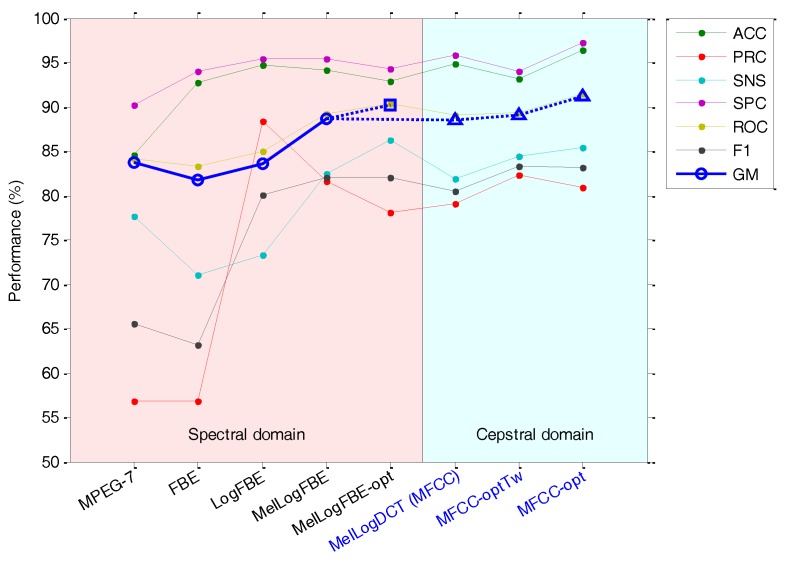
Classification performance using various alternatives for the representation of the anuran call spectrum.

Table 11 and Figure 31 summarize the classification performance metric for the eight stages between MPEG-7 and optimum MFCC. In each stage, the Geometric Mean (GM) metric was used to select the best classifier.

From these results, it can be concluded that using the log scale increases the GM by approximately two points, and using the mel scale raises this value by about five points. On the other hand, the representation of the spectrum in the cepstral domain appears to have no effect on classification performance. However, the GM can be further improved by optimizing the frame duration (with an effect of about 0.5 points) and the range of frequencies with an effect of about two points. The overall effect of the application of the steps from the FBE to the optimum MFCC increases the GM by the amount of more than nine points.

### 4.3. Reducing the Spectrum Representation Vector

In Environmental Monitoring Systems, the goal for the optimal representation of the spectrum of a sound is not only its ability to be used as features in a classification process. The size of the feature vector is also a key element because it exerts a direct impact on the storage capacity and computing power required in the WSN nodes, and also on the demand for network throughput. For this reason, reducing the number of optimal MFCC features leads to a more efficient implementation.

**Figure 32:**
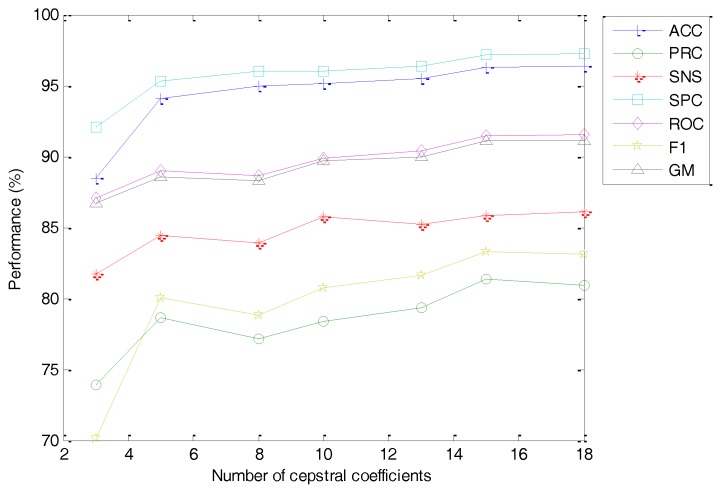
Classification performance metrics of the best classifier vs. the number of cepstral coefficients (optimal value in every option).

The classification performance metrics of the best classifier vs. the number of optimal cepstral coefficients is depicted in Figure 32. As can be observed, a smaller value of the number of cepstral coefficients (C) worsens the classifier performance metrics. This effect, however, is limited for a small reduction of C, and hence using the default number of cepstral coefficients C=13 (instead of the maximum C=20 when M=20) only means a slight reduction in performance (from ACC=96.37% to ACC=95.44%).

**Figure 33:**
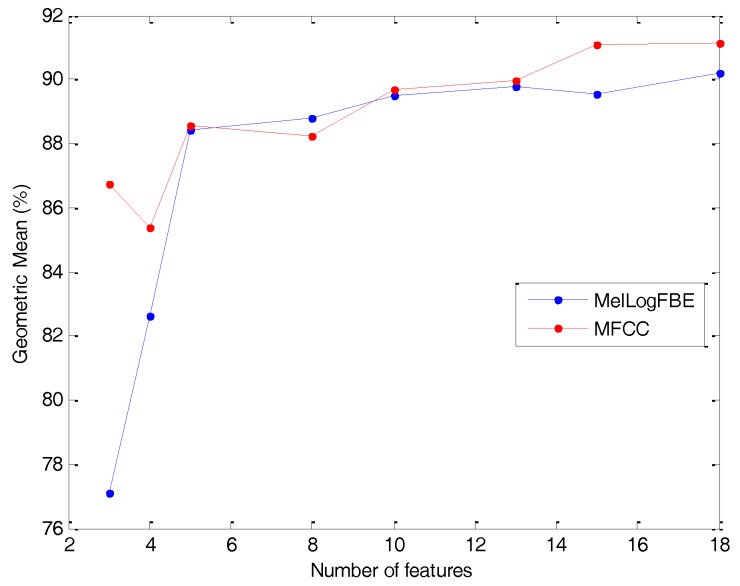
Geometric Mean of the best classifier vs. the number of features (optimal value in every option).

From the discussion in Section 4.2, it is not fully clear the advantage of using MFCC instead of the simpler FBE in log and mel scales. Both methods obtain similar classification performance metrics, although the latter requires less computation effort because it obviates the cepstral transform stage. Nevertheless, if the number of features remains a major concern, then the MFCC offers better results, as can be concluded from Figure 33, and should therefore be the extracting method selected.

## 5. Conclusions

In conclusion, for a good compromise between the classification performance and the WSN implementation considerations, the default value of 13 features should be maintained, but now with the options optimally selected. If a major reduction in the number of features is required, then the MFCC clearly outperforms the FBE.

## Figures and Tables

**Table 1:** Dataset of sounds and patterns.

Sound Class	Sound Recordings		Pattern Recordings		
	Number	Seconds	Number	Seconds (Pattern Section)	Seconds (Total Recording)
Ep. cal. mating call	369 (43%)	1853	4	13.89	20.39
Ep. cal. release call	63 (7%)	311	3	0.99	14.56
Al. ob. mating call	419 (48%)	2096	4	1.09	19.72
Al. ob. distress call	17 (2%)	83	2	3.30	9.80
Silence/Noise	-	-	-	45.20	-
Total	868	4343	13	64.47	64.47

**Table 2:** MATLAB functions supporting the various classifiers.

Classifier	Training Functions	Test Functions	Additional Functions
MinDis	-	-	
MaxLik	fitgmdist	mvnpdf	
DecTr	fitctree	predict	
kNN	fitcknn	predict	
SVM	fitcsvm	predict	
LogReg	mnrfit	mnrval	
Neur	Feedforwardnet train	net	
Discr	fitcdiscr	predict	
Bayes	fitNaiveBayes	posterior	
HMM	hmmtrain	hmmdecode	kmeanlbg disteusq

**Table 3:** Definition of the binary confusion matrix.

		Classification Class	
		Classified as Positive	Classified as Negative
**Data class**	**Positive**	TP (true positive)	FN (false negative)
	**Negative**	FP (false positive)	TN (true negative)

**Table 4:** Classification performance metrics based on the confusion matrix.

Metric	Formula	Evaluation Focus
Accuracy	$\mathrm{ACC}=\frac{\mathrm{TP}+\mathrm{TN}}{\mathrm{TP}+\mathrm{TN}+\mathrm{FP}+\mathrm{FN}}$	Overall effectiveness of a classifier
Error rate	$\mathrm{ERR}=\frac{\mathrm{FP}+\mathrm{FN}}{\mathrm{TP}+\mathrm{TN}+\mathrm{FP}+\mathrm{FN}}$	Classification error
Precision	$\mathrm{PRC}=\frac{\mathrm{TP}}{\mathrm{TP}+\mathrm{FP}}$	Class agreement of the data labels with the positive labels given by the classifier
Sensitivity	$\mathrm{SNS}=\frac{\mathrm{TP}}{\mathrm{TP}+\mathrm{FN}}$	Effectiveness of a classifier to identify positive labels
Specificity	$\mathrm{SPC}=\frac{\mathrm{TN}}{\mathrm{TN}+\mathrm{FP}}$	How effectively a classifier identifies negative labels
ROC	$ROC=\frac{\sqrt{SNS^{2}+SPC^{2}}}{\sqrt{2}}$	Combined metric based on the Receiver Operating Characteristic (ROC) space [53]
$F_{1}$ score	$F_{1}=2\frac{PRC\cdot SNS}{PRC+SNS}$	Combination of precision (PRC) and sensitivity (SNS) in a single metric
Geometric Mean	$GM=\sqrt{SNS\cdot SPC}$	Combination of sensitivity (SNS) and specificity (SPC) in a single metric

**Table 5:** Options for the extraction of features of a spectrum.

Domain	Function	Option	MPEG-7	FBE	MFCC-HTK	MFCC-opt
Time	Pre-emphasis	α	-	-	0.97	-
	Framing	Window	Hamming	Hamming	Hamming	Hamming
		$T_{w}$	30 ms	30 ms	25 ms	20 ms
		$T_{s}$	10 ms	10 ms	10 ms	10 ms
Frequency	Filter Bank Energy	$L_{f}$	64 Hz	64 Hz	300 Hz	1000 Hz
		$H_{f}$	16 kHz	16 kHz	3700 Hz	5000 Hz
		M	-	18	20	20
		Scaling	-	Linear	Mel	Mel
Quefrency	Cepstrum	Transform	-	-	DCT	DCT
		C	-	-	13	20
	Liftering	L	-	-	22	-

**Table 6:** Classification performance metrics vs. window function.

Window Function	ACC	ERR	PRC	SNS	SPC	ROC	F_1_	GM
Rectangular	91.58%	8.42%	73.12%	69.96%	92.77%	82.16%	71.51%	80.56%
Hamming	94.85%	5.15%	76.76%	81.22%	95.87%	88.49%	78.93%	88.24%

**Table 7:** Classification performance metrics vs. filter bank frequency scaling.

Filter Bank	ACC	ERR	PRC	SNS	SPC	ROC	F_1_	GM
Rectangular	94.56%	5.44%	62.57%	73.03%	96.08%	85.34%	67.40%	83.77%
Mel	94.85%	5.15%	76.76%	81.22%	95.87%	88.89%	78.93%	88.24%

**Table 8:** Classification performance metrics vs. cepstral transform.

Cepstral Transform	ACC	ERR	PRC	SNS	SPC	ROC	F_1_	GM
DFT	94.27%	5.73%	74.46%	81.17%	96.09%	88.94%	77.67%	88.31%
DCT	94.85%	5.15%	76.76%	81.22%	95.87%	88.89%	78.93%	88.24%

**Table 9:** Confusion matrix using MFCCs (optimal values in every option) and the Bayes Classifier.

		Classification Class			
		Ep. cal. Mating Call	Ep. cal. Release Call	Al. ob. Mating Call	Al. ob. Distress Call
**Data class**	Ep. cal. mating call	96.16%	0.82%	0.82%	2.19
	Ep. cal. release call	48.33%	48.33%	1.67%	1.67%
	Al. ob. mating call	2.41%	0.96%	95.90%	0.72%
	Al. ob. distress call	0%	0%	0%	100%

**Table 10:** Classification performance using different alternatives for the representation of the anuran call spectrum.

	ACC	ERR	PRC	SNS	SPC	ROC	F_1_	GM
MPEG-7 (18)	84.56%	15.44%	56.80%	77.69%	90.28%	84.22%	65.63	83.75%
FBE (18)	93.69%	7.31%	56.78%	71.05%	94.00%	83.32%	63.12%	81.72%
MFCC-HTK (13)	94.85%	5.15%	76.76%	81.22%	95.87%	88.89%	78.93%	88.24%
MFCC-opt (13)	95.44%	4.56%	79.38%	84.00%	96.34%	90.38%	81.63%	89.96%
MFCC-opt (20)	96.37%	3.63%	81.28%	85.10%	97.21%	91.35%	83.15%	90.95%

**Table 11:** Classification performance using various alternatives for the representation of the anuran call spectrum.

	ACC	ERR	PRC	SNS	SPC	ROC	F_1_	GM
MPEG-7	84.56%	15.44%	56.80%	77.69%	90.28%	84.22%	65.63	83.75%
FBE	93.69%	7.31%	56.78%	71.05%	94.00%	83.32%	63.12%	81.72%
LogFBE	94.74%	5.26%	88.31%	73.31%	95.37%	85.06%	80.12%	83.62%
MelLogFBE	94.15%	5.85%	81.58%	82.52%	95.41%	89.19%	82.05%	88.73%
MelLogFBE-opt	92.87%	7.13%	78.16%	86.25%	94.31%	90.37%	82.00%	90.19%
MelLogDCT	94.85%	5.15%	79.15%	81.91%	95.78%	89.12%	80.51%	88.58%
MFCC-optTw	93.10%	6.90%	82.31%	84.44%	94.02%	89.36%	83.36%	89.10%
MFCC-opt	96.37%	3.63%	81.78%	85.09%	91.17%	91.33%	83.40	90.93%
